# A conserved histidine in switch-II of EF-G moderates release of inorganic phosphate

**DOI:** 10.1038/srep12970

**Published:** 2015-08-12

**Authors:** Ravi Kiran Koripella, Mikael Holm, Daniel Dourado, Chandra Sekhar Mandava, Samuel Flores, Suparna Sanyal

**Affiliations:** 1 Department of Cell and Molecular Biology, Uppsala University, Box-596, BMC, 75124, Uppsala, Sweden

## Abstract

Elongation factor G (EF-G), a translational GTPase responsible for tRNA-mRNA translocation possesses a conserved histidine (H91 in *Escherichia coli*) at the apex of switch-II, which has been implicated in GTPase activation and GTP hydrolysis. While H91A, H91R and H91E mutants showed different degrees of defect in ribosome associated GTP hydrolysis, H91Q behaved like the WT. However, all these mutants, including H91Q, are much more defective in inorganic phosphate (Pi) release, thereby suggesting that H91 facilitates Pi release. In crystal structures of the ribosome bound EF-G•GTP a tight coupling between H91 and the γ-phosphate of GTP can be seen. Following GTP hydrolysis, H91 flips ~140° in the opposite direction, probably with Pi still coupled to it. This, we suggest, promotes Pi to detach from GDP and reach the inter-domain space of EF-G, which constitutes an exit path for the Pi. Molecular dynamics simulations are consistent with this hypothesis and demonstrate a vital role of an Mg^2+^ ion in the process.

Elongation factor G (EF-G) belongs to the subfamily of translational G-proteins in the GTPase superfamily^1^. All G-proteins share a nucleotide binding G domain, which contains distinct and highly conserved elements (G1-G5)^1^,^2^. The G3 sequence motif, ‘switch II’, is highly flexible and contains a DXXG sequence^1^,^2^. It has been suggested that a conserved glutamine (Q) immediately after the glycine of the DXXG motif, positioned at the tip of switch II is crucial for GTP hydrolysis in Ras-like G proteins^2^,^3^. However, in all translational GTPases this glutamine is replaced by a histidine^1^ (Fig. 1a). In EF-G from *Escherichia coli* (*E. coli*), this histidine is H91 based on its residue number on the mature EF-G chain after removal of the starting f-Met (Fig. 1b). The corresponding histidine on EF-Tu is H84; high resolution structures of EF-G and EF-Tu on the ribosome with GTP analogues place H91 and H84 in similar conformations, pointing towards the γ-phosphate of GTP through the hydrophobic gate^4^,^5^,^6^.

The role of this His in GTP hydrolysis by EF-Tu and EF-G has been extensively studied. Several groups have reported that mutations of H84 inhibit ribosome stimulated GTP hydrolysis by EF-Tu^7^,^8^,^9^,^10^. Similarly, the H91A mutation in EF-G has been claimed to be completely^11^,^12^ or significantly^13^ impaired in GTP hydrolysis. Based on a crystal structure of EF-Tu•GDPNP on the ribosome it has been proposed that H84 acts as a catalytic residue that deprotonates a water molecule for in-line attack on the bond between β- and γ-phosphates of GTP^6^. This proposal, however, has been challenged and an alternative mechanism where H84 plays an indirect stabilizing role for the catalytic water has been suggested^14^,^15^. Thus, the current understanding of the field is that this His may not be catalytic for GTP hydrolysis, but is important for GTPase activation of the translational GTPases on the ribosome^16^,^17^.

Compared to GTP hydrolysis, fewer studies have been conducted to understand the mechanism of Pi release and its role in EF-G functions. It has been shown that, restriction of the inter-domain movements in EF-G by introducing a disulphide cross-link between domains I and V renders the factor completely inactive in translocation, but leaves both GTP hydrolysis and Pi release unaffected^18^. Later studies indicated that Pi release is not a pre-requisite for translocation and might occur through an independent, parallel pathway^19^. However, Pi release was shown to be essential for EF-G release from the ribosome^20^ and also, for EF-G function in ribosome disassembly^21^. The ribosomal stalk protein L7/L12 has been proposed to be involved in the Pi release process, but the mechanism is yet to be clarified^20^.

Recent high resolution structures of EF-G on the ribosome in complex with either GTP analogs (GDPCP and GDPNP)^4^,^5^, or GDP and fusidic acid (FA)^22^,^23^,^24^ show a dramatic change in the orientation of H91 upon GTP hydrolysis. Compared to the structures with the GTP analogue GDPCP or GDPNP, where the side chain of H91 points towards the γ-phosphate and remains tightly coupled via a salt bridge^4^,^5^, in structures with GDP and fusidic acid, it is rotated by 140° in the opposite direction. This striking observation prompted us to think that the ‘flip’ of the H91 following GTP hydrolysis might provide an efficient mechanism for Pi release. To test this idea, we created several mutant EF-Gs by replacing H91 with a series of amino acids (H91A, H91E, H91Q and H91R) and studied these mutant EF-Gs in GTP hydrolysis and Pi release with pre-steady state kinetics. As a control, we have mutated another highly conserved residue on switch II, Phe94 to Leu (F94L) and tested this mutant in parallel. F94 is known to be important for conformational changes in EF-G on and off the ribosome^25^,^26^ and mutation of F94 confers high level FA resistance to the bacteria, but also leads to loss of fitness^25^,^27^.

Our results show that the degree of defect in GTP hydrolysis varies in the H91 mutants with the exception of H91Q, which is not impaired in GTP hydrolysis. However, all the H91 mutants are defective in Pi release. Moreover, the relative decrease in the rate of Pi release is always greater than that in GTP hydrolysis. We also performed molecular dynamics (MD) simulations, which show that H91 remains coupled to Pi even after GTP hydrolysis. Combining our results with information from high resolution structures we propose that by ‘flipping’ after GTP hydrolysis, H91 guides the release of Pi by positioning it in an inter-domain space of EF-G, that constitutes an unhindered exit path for the Pi.

## Results

### The EF-G variants show similar affinity to (mant)-GTP

We have estimated the affinity of the EF-G variants (WT, F94L, H91A, H91E, H91Q and H91R) for mant-GTP, a fluorescent GTP analogue^28^. *K*_D_ values were estimated by plotting the relative increase in mant fluorescence against the concentration of mant-GTP (Fig. S1). The *K*_D_ values were similar for all six EF-G variants (Table 1); 8.2 ± 0.3 μM for the WT, 8.5 ± 1.9 μM for F94L, 10.6 ± 0.3 μM for H91A, 8.1 ± 0.3 μM for H91E, 13.4 ± 1.3 μM for H91Q and 14.3 ± 1.8 μM for H91R EF-G. Thus, the H91 mutants and F94L display similar proficiency in binding mant-GTP and thus, by inference native GTP.

### Intrinsic GTP hydrolysis by EF-G is unaffected by the H91 and F94 mutations

**Figure 2:**
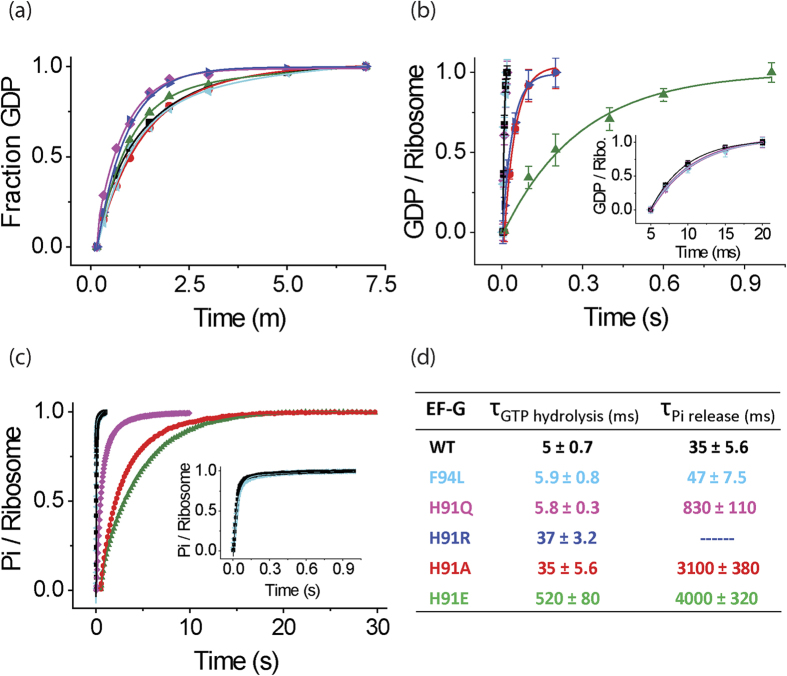
GTP hydrolysis and Pi release by the EF-G variants. Time course of GTP hydrolysis by WT (black), F94L (cyan), H91A (red), H91E (green), H91Q (magenta) and H91R (blue) EF-Gs in the absence (**a**) and presence of 70S ribosomes (**b**) measured under single turnover conditions. The amount of [^3^H] GDP formed is plotted against time and fitted with a single exponential function to estimate the rate constant of GTP hydrolysis. Error bars represent standard deviations obtained from three independent experiments. (**c**) Single turnover Pi release in the presence of 70S ribosomes studied by fluorescence increase of MDCC-PBP. The traces are fitted with single exponential function. Inset in (**b**) shows WT, F94L and H91Q data while inset in (**c**) shows WT and F94L data on a shorter time scale. (**d**) Mean times (τ) for GTP hydrolysis and Pi release estimated from the experiments in b and c.

We studied the spontaneous GTPase activity of the EF-G variants by monitoring the time course of GTP hydrolysis in the absence of 70S ribosomes. For that, EF-G in excess was mixed with [^3^H]GTP at 37 °C and the reaction was quenched with 25% formic acid at different time points. The amount of GDP formed was estimated from the ratio of the peak area of [^3^H]GDP and [^3^H]GTP as separated on a MonoQ column coupled to HPLC. The rate constant for the intrinsic GTPase activity of WT EF-G was estimated as 0.013 ± 0.003 s^−1^, in close agreement with earlier reports^13^,^26^. Interestingly, none of the mutations affected the rate of intrinsic GTP hydrolysis. The rate constants for all EF-G variants were estimated as 0.014 ± 0.003 s^−1^ for F94L, 0.012 ± 0.001 s^−1^ for H91A, 0.014 ± 0.004 s^−1^ for H91E, 0.02 ± 0.006 s^−1^ for H91Q and 0.019 ± 0.004 s^−1^ for H91R (Fig. 2a, Table 1). Thus, in the absence of ribosome, GTP hydrolysis by EF-G does not involve H91. This is highly similar to a recent result with H84 mutations in EF-Tu, which did not show any defect in intrinsic GTPase activity^29^.

### H91 mutated EF-Gs show different degrees of defect in ribosome-stimulated GTP hydrolysis

GTP hydrolysis experiments in the presence of 70S ribosomes were performed in a quench-flow instrument. As shown in earlier reports, the GTPase activity of EF-G was significantly stimulated by the ribosome^13^,^26^,^30^. The WT EF-G hydrolyzed GTP on the ribosome with a single turnover rate constant of *k*_GTP WT_ = 202 ± 29 s^−1^, which is about 15 000 times larger than the rate constant of intrinsic GTP hydrolysis, as also reported earlier^31^. Mutants H91Q (*k*_GTP H91Q_ = 174 ± 12 s^−1^) and the control mutation F94L (*k*_GTP F94L_ = 170 ± 28 s^−1^) hydrolyzed GTP with rate constants comparable to that of WT EF-G (Fig. 2b, Table 1). In contrast, the H91A, H91E and H91R EF-Gs were defective in GTP hydrolysis, although the degree of defect varied. Mutants H91A and H91R showed a seven fold decrease in the rate constant compared to the WT (*k*_GTP H91A_ = 28 ± 4.5 s^−1^ and *k*_GTP H91R_ = 27 ± 2.5 s^−1^), while H91E showed a rate reduction by a factor of 100 (*k*_GTP H91E_ = 1.9 ± 0.3 s^−1^) (Fig. 2b, Table 1). The mean time analysis (i.e. inverse of rate constants) shows that compared to 5 ± 0.7 ms for the WT, the H91A and H91R take 35 ± 5.6 ms and 37 ± 3.2 ms respectively, and H91E takes 520 ± 82 ms to hydrolyze one GTP on the ribosome (Fig. 2d). Since the H91 mutant EF-Gs show similar affinity to GTP as the WT, the defect in ribosome stimulated GTP hydrolysis must have originated from their interaction with the ribosome.

### H91 mutants show larger defects in Pi release than in GTP hydrolysis

Single turnover Pi release was studied using stopped-flow, with MDCC labelled phosphate binding protein (PBP-MDCC), the fluorescence of which increases instantaneously upon Pi binding^32^,^33^. In the presence of the ribosome, WT EF-G released Pi with a rate constant of *k*_Pi WT_ = 28 ± 4.7 s^−1^ (Fig. 2c, Table 1), which is much slower than GTP hydrolysis. In comparison to the WT, except for the control mutation F94L, which did not show any defect in Pi release (*k*_Pi F94L_ = 21 ± 3.5 s^−1^), all the H91 mutants were much slower in Pi release (Fig. 2c, Table 1). The H91Q mutant, which did not show any defect in GTP hydrolysis, was over 20fold slower than WT EF-G in releasing Pi (*k*_Pi H91Q_ = 1.2 ± 0.16 s^−1^). The other two mutants, H91A and H91E released Pi with rate constants *k*_Pi H91A_ = 0.32 ± 0.04 s^−1^ and *k*_Pi H91E_ 0.25 ± 0.02 s^−1^ respectively. Pi release by H91R EF-G could not be measured. This is probably due to very slow release of Pi, which is either beyond the fluorescence life-time of PBP-MDCC or is readily removed by the phosphate mop (*see* Materials and Methods) present in the reaction. These results suggest that H91 plays an important role in Pi release from EF-G.

### Molecular dynamics simulations are consistent with the role of H91 in Pi release

**Figure 3:**
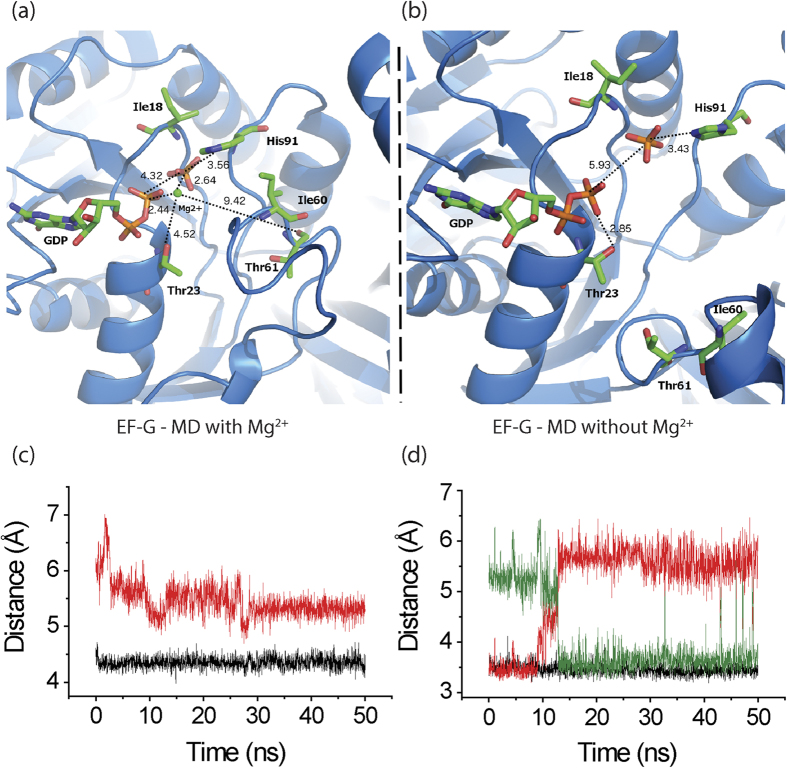
MD simulation results. (**a**,**b**) Structural views of the guanine-nucleotide binding pocket and switch II of EF-G with (**a**) and without (**b**) Mg^2+^ ion (green sphere) from the MD simulation experiments. The initial models are built using MacroMoleculeBuilder (MMB)^34^, on the basis of the crystal structure of EF-G•GDPCP bound to the ribosome (PDB: 4CR1)^4^. Both models represent the scenario immediately after GTP hydrolysis but before Pi release. We did not observe any relevant structural differences in the replica MDs. (**c**,**d**) Time trace for the distances between Pi and β-phosphate (**c**) and Pi and H91 (either Nδ_1_ or N_ε2_) (**d**) estimated from MD structures with (black trace) or without (red/green trace) Mg^2+^ ion. The red and the green traces in (**d**) are distances involving N_δ1_ or N_ε2_ atoms of the H91 side chain, respectively.

Based on the crystal structure of the ribosome bound to EF-G•GTP (PDB: 4CR1)^4^, we have built and equilibrated an initial model of EF-G bound to GDP and Pi using MacroMoleculeBuilder (MMB)^34^ and with MD simulations^35^. This model essentially mimics the situation after GTP hydrolysis but before Pi release. In MD, the distance between Pi and the β-phosphate was 4.3 Å (Fig. 3a,c), while between H91 (N_δ1_) and Pi it was 3.6 Å (Fig. 3a,d); this did not differ significantly from what was observed with MMB. In addition, a strong coordination between Pi and Mg^2+^ was observed in both MMB and MD runs, suggesting that the Mg^2+^ stabilizes Pi even after GTP hydrolysis.

Close comparison of the ribosome bound EF-G•GTP structures (PDB: 4CR1, 4JUW)^4^,^5^ with the EF-G•GDP structures showed disappearance (PDB: 4KDA)^24^ or major repositioning of the Mg^2+^ with respect to β-phosphate and Pi (PDB: 2WRI)^22^ (Fig. S2). This suggests that in order for the Pi to be released the Mg^2+^ needs to be repositioned. To test this hypothesis, another model was built without the Mg^2+^ and equilibrated in MMB. Then the model was relaxed with MD as the former one (Fig. 3b). We saw an immediate increase in distance between β-phosphate and Pi to ~5.9 Å (Fig. 3b,c). In contrast, the distance between Pi and H91 (either N_δ1_ or N_ε2_) remained unchanged at ~3.5 Å (Fig. 3b,d), suggesting that the Pi was strongly coupled with H91. Thus, H91 and Pi could together move towards the open GDP-bound configuration. This is consistent with the suggestion from the biochemical data that H91 facilitates Pi release.

## Discussion

**Figure 4:**
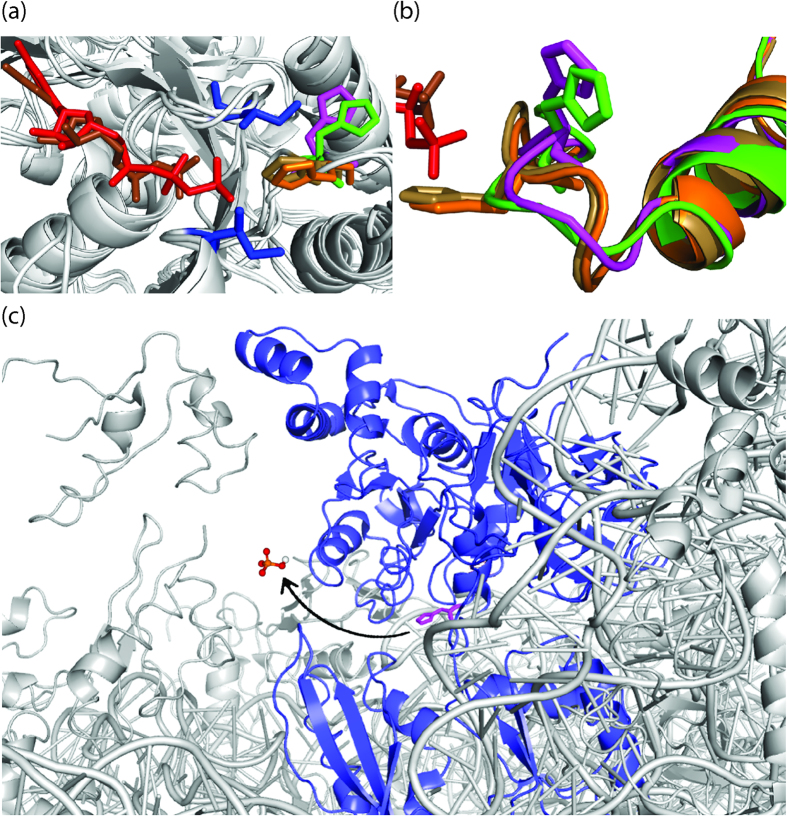
The orientations of the H91 side chain and the exit path for Pi. (**a**) Superimposition of the crystal structures of EF-Gs (*Thermus thermophilus*) showing orientation of H91 side chain (H87 in *T. thermophilus*); with GDPCP (PDBs: 4JUW^5^ in orange and 4CR1^4^ in sand) and with GDP and fusidic acid (PDBs: 4KDA^24^ in green and 2WRI^22^ in magenta). The “hydrophobic gate” formed by residues Ile20 and Ile63 are shown in blue and the bound GDP and GDPCP are shown in brown and red respectively. (**b**) Close-up view of the switch II loop with its conserved H91, color code as in (**a**) shows that the whole switch-II loop following the superimposed α helix changes its orientation in the GTP/GDP state. (**c**) The potential exit path of Pi (modelled in the crystal structure (PDB: 2WRI)^22^ through the inter-domain space of EF-G•GDP (blue) on the ribosome (grey). It can be observed that the Pi exit path (marked with an arrow) in front of H91 (magenta) is not hindered by any ribosomal components.

Our results show that GTP hydrolysis by EF-G gets strongly stimulated by the ribosome (Table 1)^26^, suggesting that the ribosomal components act as the GTPase activating factor for EF-G^30^,^36^. This is in agreement with the previous results and also similar to EF-Tu, where the rate of GTP hydrolysis has been reported to increase by a million fold upon binding to the ribosome^29^. In structural terms, the activation is probably achieved by proper positioning of the two switch regions of EF-G in relation to GTP and also by opening the so called ‘hydrophobic gate’ composed of the two hydrophobic amino acids Ile18 and Ile60 (*E. coli* numbers) (Fig. 4a). In crystal structures of EF-G with GTP analogues, H91 is directed towards the γ-phosphate with an average distance of 3.6 Å between them, which is typical for a salt bridge (Fig. 4a)^4^,^5^. Although the exact role of H91 in GTP hydrolysis cannot be deduced from these structures, a close coordination of H91 and the γ-phosphate is evident.

In this work, we have characterized four H91 mutant EF-Gs for GTP hydrolysis and Pi release and compared those with WT and F94L EF-Gs. All of these EF-G mutants have similar affinity to GTP in solution and the rate of intrinsic GTP hydrolysis is not affected by any of these mutations. This result, similar to EF-Tu suggests that the spontaneous GTP hydrolysis by EF-G does not depend on H91^29^. In a recent study, a monovalent cation far from H84 has been suggested as the catalytic cofactor for GTP hydrolysis in ribosome unbound EF-Tu^37^ and likely to be the same in EF-G. It should be noted that in the free form of EF-G, H91 points away from the γ-phosphate of GTP (PDB: 2J7K)^38^, which explains why H91 is not involved in intrinsic GTPase activity of EF-G. However, on the ribosome, the mutated EF-Gs display distinct behavior. The largest defect in ribosome stimulated GTP hydrolysis was identified for the H91E mutant. This result is not unexpected, since in this case the bulky positive side chain of His is replaced by a negative side chain of Glu. This swap will not only destroy the coupling between the amino acid and the γ-phosphate, but may also alter the charge distribution and the intramolecular interactions in the GTP binding pocket. In contrast, no defect was observed for the H91Q mutant. This is also expected since Gln is present instead of His in all G-proteins except the translational GTPases. When side chains are compared, the NH_2_ group of Gln can easily be superimposed on the NH group of His, which is actually involved in coupling with the γ-phosphate. Thus, it follows naturally that the H91Q mutant acts like the WT in GTP hydrolysis. Similar results have also been reported with the corresponding mutation in EF-Tu (H84Q), which showed only small to moderate reduction in the GTPase activity^9^,^10^. However, a recent report on H84Q mutation of *E. coli* EF-Tu shows about a 4000 fold decrease in the rate of GTP hydrolysis^29^, which contradicts the earlier results. Further investigation will be needed to solve the controversy in the rate of GTP hydrolysis by H84Q EF-Tu.

The H91A and H91R mutants are about seven fold slower than the WT in ribosome stimulated GTP hydrolysis. In these two cases, the positive side chain of His is replaced either with a small nonpolar side chain (A) or a longer basic side chain (R). In H91A, the lack of proper orientation of the β and γ-phosphate of GTP can be expected due to absence of the coupling between the γ-phosphate and the nonpolar small side chain of Ala. The H91R mutant, in contrast, might have additional coupling with multiple phosphates of GTP due to the occurrence of three N atoms in its side chain. It should be mentioned that the H91A EF-G was previously reported as completely inactive in GTP hydrolysis^11^. This apparent discrepancy, we think, may arise from differences in the sample preparation and experimental conditions.

Compared to the mean time of GTP hydrolysis by WT EF-G (~5 ms) Pi release takes much longer time (~35 ms) (Fig. 2d), which suggests that Pi remains trapped in the nucleotide binding pocket even after GTP is hydrolyzed. Interestingly, the Pi retention time is significantly longer than the WT for all the four H91 mutants (lowest 4000 ms), while for the F94L mutant it is similar to the WT (Fig. 2d). Moreover, comparison of the mean times of GTP hydrolysis and Pi release clearly demonstrates a much bigger defect in Pi release for all the H91 mutants than in GTP hydrolysis. Thus, our results suggest a direct involvement of H91 in Pi release.

To understand the structural basis of Pi release we compared the crystal structures of EF-G bound to GTP analogues (PDB: 4CR1, 4JUW)^4^,^5^ and GDP, both on the ribosome (PDB: 2WRI, 4KDA)^22^,^24^ and in the free state (PDB: 1FNM, 2BM0, 4M1K, 4MYU)^39^,^40^. While the most obvious change can be seen in the orientation of the H91 side chain, which ‘flipped’ almost 130°–140° (Fig. 4a) and consequently altered the conformation of the entire switch II loop (Fig. 4b), several other changes were visible. Detailed inspection of the GTP binding site dragged our attention to a Mg^2+^ ion coordinating with both the β- and the γ-phosphates of GTP. Interestingly, in the EF-G•GDP structures this Mg^2+^ was either absent (PDB: 4KDA)^24^ or its position varied a lot (PDB: 2WRI)^22^ (Fig. S2). This analysis suggested that displacement of the Mg^2+^ might be necessary for Pi release. Our MD simulations indeed support this hypothesis. When the EF-G•GDP•Pi model contained the Mg^2+^ ion, no change in the interatomic distances was observed during the MD run (Fig. 3a). In contrast, an immediate increase in the distance between the β-phosphate and Pi was noticed when the model was built without the Mg^2+^ (Fig. 3b). However, H91 remained constantly coupled with the Pi although the His side chain might undergo a rotation thereby switching the coupling from the N_δ1_ to the N_ε2_ atom. Thus we propose that the tight coupling of Pi to H91 and the ‘flipping’ movement of H91are the two key determinants of Pi release.

The lack of coupling with Pi in H91A and H91E EF-Gs renders them significantly defective in Pi release. On the contrary, perhaps a too strong coupling with the Arg side chain in H91R makes Pi release so slow that it could not be measured under our experimental conditions. Most interestingly, the H91Q mutant, which is not impaired in GTP hydrolysis showed a more than 20 fold defect in Pi release. It suggests that although EF-G can attain the optimal conformation for GTP hydrolysis with Gln instead of His, the bulky side chain of His is instrumental in changing the conformation of switch II required for Pi release (Fig. 4b). This view is further supported by the observation that in the flipped conformation (GDP state), the H91 side chain opens into a tunnel which runs through the inter domain space of EF-G^22^,^24^. Since Pi remains coupled with H91 after GTP hydrolysis, most likely it follows H91 and thereby reaches this tunnel, which constitutes the exit path for the Pi (Fig. 4c). This path is free from steric obstructions even on the ribosome (Fig. 4c). Thus, we infer that the tight coordination of Pi with H91 and the ‘flipping’ of the H91 side chain are the key determinants for efficient Pi release. This mechanism may have general significance as other translational GTPases (e.g. EF-Tu) possess a His corresponding to H91, which undergoes similar structural transitions.

## Materials and Methods

### Mutagenesis of EF-G and protein purification

The WT *fusA* gene form *E. coli* cloned into plasmid pET24b with a C-terminal hexa-histidine tag was a kind gift from Kevin S. Wilson, Oklahoma state University, USA^26^. Using this construct as a template and following standard protocols for site-directed mutagenesis, the H91 residue was mutated to alanine (A), Glutamine (Q), Arginine (R) and glutamic acid (E), resulting into four mutant EF-Gs, H91A, H91Q, H91R and H91E respectively. In a similar way, residue F94 was mutated to Leucine (L), creating F94L mutant. The mutations were confirmed by DNA sequencing. Over-expression and purification of the WT and mutant EF-Gs were carried out as described earlier^25^, by using Ni^2+^ affinity chromatography followed by size exclusion. The homogeneity of the WT and mutant EF-Gs was confirmed by SDS-Page followed by mass spectrometric analysis.

### Buffers and components

All experiments were performed in HEPES polymix buffer (pH 7.5) containing 5 mM NH_4_Cl, 5 mM Mg(OAc)_2_, 100 mM KCl, 0.5 mM CaCl_2_, 8 mM putrescine, 1 mM spermidine, 5 mM HEPES and 1 mM dithioerythritol (DTE) at 37 °C with a free Mg^2+^ concentration of around 1.5 mM. MRE600 70S ribosomes were purified according to the published methods^33^,^41^. Nucleotide triphosphates were from GE-Healthcare. Plasmid construct for phosphate binding protein (PBP) was a kind gift from Prof. Martin Webb, London. The purification and 7-Diethylamino-3-((((2-Maleimidyl) ethyl)amino) carbonyl)coumarin (MDCC) labelling of PBP was done according to the published protocol^32^.

### Single-round GTP hydrolysis

Intrinsic GTP hydrolysis by EF-G was measured under single turnover condition by mixing equal volumes of [^3^H]GTP(15 μM) and EF-G (30 μM) manually at 37 °C; the reaction was quenched at different time points with 25% formic acid. The amount of [^3^H]GDP formed was estimated by separating the [^3^H]GTP and [^3^H]GDP fractions on a MonoQ column attached to a HPLC system.

The GTPase activity of the EF-Gs on the ribosome was measured in a similar way by mixing vacant 70S ribosomes (3 μM) and [^3^H] GTP (15 μM) (in one mix) with EF-G (15 μM) in a quench-flow device (RQF-3 KinTek Corp.) at 37 °C. In order to estimate the single turnover GTP hydrolysis rate, data points only from the initial fast phase were used. The rate constants of GTP hydrolysis (*k*_*GTP*_) was obtained by fitting the data with a single exponential function using ORIGIN 8.0 (Originlab Corporation). The mean time τ_*GTP*_ was estimated as (1/*k*_*GTP*_).

### Single-round Pi release

The release of inorganic phosphate from EF-G after GTP hydrolysis was measured in a stopped flow instrument (Applied Photophysics SX20) by monitoring the fluorescence of MDCC-PBP that shows an immediate increase in fluorescence upon binding to Pi^32^,^33^. Mix A containing 70S ribosomes (2 μM) and GTP (100 μM) was rapidly mixed with mix B containing EF-G (20 μM) and MDCC-PBP (1 μM) after preincubating both mixes for 10 minutes at 37 °C. Both mixes also contained a phosphate mop comprising 7-methylguanosine (300 μM) and purine nucleoside phosphorylase (0.1 unit/μl). The rate constants of Pi release (*k*_*Pi*_) was estimated by fitting the fluorescence time traces with a single exponential function using Origin 8.0 (Originlab Corp.). The mean time for Pi release (τ_*Pi*_) was estimated as (1/*k*_*Pi*_).

### Measurement of affinity for GTP

The equilibrium dissociation constant (*K*_D_) for GTP to EF-G was estimated using a fluorescent analog of GTP called mant-GTP ((2′, 3′)-*O*-*N*-methylanthraniloyl-GTP). When exited at 290 nm, fluorescence resonance energy transfer (FRET) occurs between the Trp residues present in the proximity of the nucleotide binding pocket in EF-G and the mant group of mant-GTP, thereby leading to increase in its fluorescence^28^. EF-G (2 μM) was mixed with increasing amounts of mant-GTP (5–60 μM) at 37 °C and the fluorescence change at 445 nm was recorded in a fluorimeter (HITACHI F-7000). The difference in the FRET signal with and without EF-G was plotted against the concentration of mant-GTP and the *K*_D_ was estimated by fitting of a hyperbolic function using ORIGIN 8.0 (Originlab Corporation).

### Modelling of EF-G•GDP – Pi and Molecular Dynamics simulations

We built and equilibrated an initial model of EF-G bound to GDP and Pi using MMB^34^ based on the crystal structure of the ribosome bound to EF-G•GTP (PDB: 4CR1)^4^, as a reduced-dimensionality prototype simulation prior to full MD. During equilibration, EF-G residues 83-91, Mg^2+^, β-phosphate of GDP and Pi were flexible. We placed a small water droplet restrained to a ~1.6 nm radius about H91. The PARM99 force field^35^ was applied to the water and all atoms within 1.2 nm of the flexible regions. A strong coordination between Pi and Mg^2+^ was observed, in addition to coordination between H91 and Pi. To understand the role of the Mg^2+^, another model without Mg^2+^ was built and similarly equilibrated in MMB. For MD simulation, GDP parameters (charges, dihedrals, angles, bonds, and van der Waals parameters) were based on Meagher *et al.*^42^. For Pi, the atomic point charges were calculated with the GAUSSIAN 09^43^ at the HF/6-31G* level, while the other parameters were obtained with general Amber force field (GAFF)^35^. The model with Mg^2+^ was solvated with 45347 single point charge (SPC) waters while the model without Mg^2+^ was solvated with 46144 SPC waters. Both systems were submitted to 100 steps of steep descent energy minimization to remove bad contacts between the solvent and the protein, followed by 200 ps of restrained MD simulation to relax the water molecules. Finally, for both models, two random initial velocity 50 ns MD runs were performed using a time step of 0.002 ps. Periodic boundary conditions were used in a NPT ensemble (v-rescale thermostat^44^ and Parrinello-Rahman barostat^45^ - τ_T_ = 0.1 ps, T_ref_ = 300 K, P_ref_ = 1 bar). The Particle Mesh Ewald (PME)^46^ method was applied with a cut-off of 1.0 nm. A twin range cut-off with neighbor list cut-off 1.0 was used for the van der Waals interactions. All simulations and consequent analyses were carried out using the Gromacs 4.6.5 software package conjugated with the PARM99 force field^35^,^47^.

## Additional Information

**How to cite this article**: Koripella, R. K. *et al.* A conserved histidine in switch-II of EF-G moderates release of inorganic phosphate. *Sci. Rep.* **5**, 12970; doi: 10.1038/srep12970 (2015).

## Supplementary Material

Supplementary Information

## Figures and Tables

**Figure 1:**
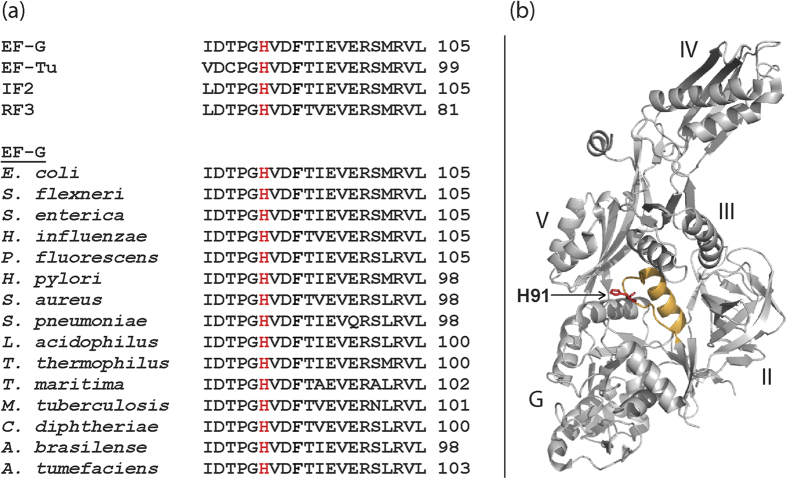
Sequence conservation and location of H91 in EF-G. (**a**) Comparison of the amino acid sequences of the switch II loop containing DXXG (G3) motif in four translational GTPases from *E. coli,* and EF-G from various bacteria (*Escherichia coli, Shigella flexneri, Salmonella enterica, Haemophilus influenzae, Pseudomonas fluorescens, Helicobacter pylori, Staphylococcus aureus, Streptococcus pneumoniae, Lactobacillus acidophilus, Thermus thermophilus, Thermotoga maritime, Mycobacterium tuberculosis, Corynebacterium diphtheriae, Azospirillum brasilense and Agrobacterium tumefaciens* listed from top). The conserved H91 residue is marked in red. (**b**) The structure of *Thermus thermophilus* EF-G (PDB ID: 1FNM) with H87 (corresponding to H91 in *E. coli*) marked in red on the apex of switch II (in gold).

**Table 1:** The rate constants of single turnover GTP hydrolysis and Pi release by the EF-G variants in the absence (−70S) and presence (+70S) of the ribosome.

**EF-G**	***K*_D GTP_(μM)**	***k*_GTP_ (s^−1^) −70S**	***k*_GTP_ (s^−1^) +70S**	***k*_Pi_ (s^−1^) +70S**
WT	8.2 ± 0.3	0.013 ± 0.003	202 ± 29	28 ± 4.7
F94L	8.5 ± 1.9	0.014 ± 0.003	170 ± 28	21 ± 3.5
H91Q	13.4 ± 1.3	0.02 ± 0.006	174 ± 12	1.2 ± 0.16
H91R	14.3 ± 1.8	0.019 ± 0.004	27 ± 2.5	-------
H91A	10.6 ± 0.3	0.012 ± 0.001	28 ± 4.5	0.32 ± 0.04
H91E	8.1 ± 0.3	0.013 ± 0.003	1.9 ± 0.3	0.25 ± 0.02

The first column represents the affinity of the EF-Gs for (mant) GTP. Uncertainties represent standard deviations obtained from three independent experiments.
