# Supplementary material for: A conserved histidine in switch-II of EF-G moderates release of inorganic phosphate
Source: Sci Rep. 2015 Aug 12;5:12970. doi: 10.1038/srep12970 (PMC4532990; doi:10.1038/srep12970)
Supplement: Supplementary Information [file srep12970-s1.pdf]

Supplementary Information

A conserved histidine in switch-II of EF-G moderates release of inorganic phosphate

Ravi Kiran Koripella, Mikael Holm, Daniel Dourado, Chandra Sekhar Mandava, Samuel Flores and Suparna Sanyal*

Department of Cell and Molecular Biology, Uppsala University, Box-596, BMC, 75124, Uppsala, Sweden

*Correspondence: suparna.sanyal@icm.uu.se, 0046-18-471 4220

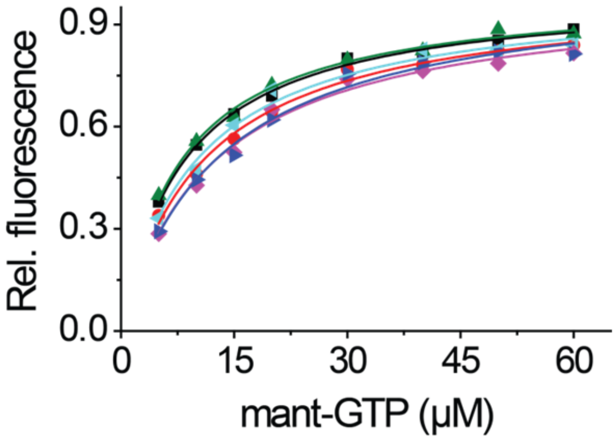

Figure S1. Affinity of EF-G for mant-GTP. The affinity of various EF-G variants WT (black), F94L (cyan), H91A (red), H91E (green), H91Q (magenta) and H91R (blue) for mant-GTP was measured by titrating mant-GTP against a fixed concentration of EF-G. The relative fluorescence increase from saturation binding of EF-G was plotted against concentrations of mant-GTP. The data points were fitted using a hyperbolic equation to estimate the K_D values.

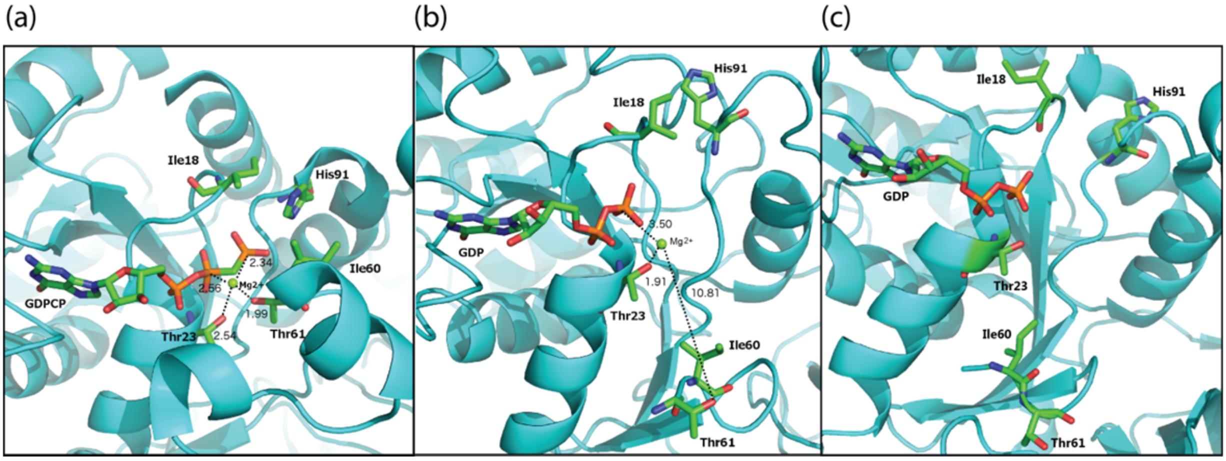

Figure S2. Position of the Mg^{2+} ion in the crystal structures of EF-G on the ribosome. (a) with GDPCP (PDB: 4CR1) [1], (b-c) with GDP (and fusidic acid); PDBs 2WRI (b) [2]and 4KDA (c) [3], respectively. The structure in (c) lacks the Mg^{2+} ion. Other important residues and intramolecular interactions are shown with labels or dotted lines.

References

1. Chen, Y., Feng, S., Kumar, V., Ero, R. & Gao, Y.G. Structure of EF-G-ribosome complex in a pretranslocation state. *Nat Struct Mol Biol* **20**, 1077-84 (2013).
2. Gao, Y.G. et al. The structure of the ribosome with elongation factor G trapped in the posttranslocational state. *Science* **326**, 694-9 (2009).
3. Zhou, J., Lancaster, L., Donohue, J.P. & Noller, H.F. Crystal structures of EF-G-ribosome complexes trapped in intermediate states of translocation. *Science* **340**, 1236086 (2013).
